# MR imaging biomarkers evaluating vascular normalization window after anti-vessel treatment

**DOI:** 10.18632/oncotarget.22600

**Published:** 2017-11-21

**Authors:** Jun Yang, Chengde Liao, Yifan Liu, Guangjun Yang, Tengfei Ke, Yingying Ding, Qinqing Li

**Affiliations:** ^1^ Department of Radiology, The Third Affiliated Hospital of Kunming Medical University, Yunnan Cancer Hospital, Kunming 650118, Yunnan, P.R. China

**Keywords:** anti-angiogenic therapy, vascular normalization window, glioma, DCE-MRI, IVIM-MRI

## Abstract

The beginning and the end of the vascular normalization window are not clear in response to anti-angiogenic therapies. We used dynamic contrast-enhanced MRI (DCE-MRI) and intravoxel incoherent motion MRI (IVIM-MRI) to noninvasively evaluate the vascular normalization window. MRI was performed five times: before treatment and on the second, fourth, sixth and eighth days of treatment. Quantitative perfusion parameters were calculated at each time point, including the volume transfer coefficient (Ktrans), reverse transfer constant (Kep), pseudodiffusion coefficient (D^*^) and perfusion fraction (f). Tumors were evaluated for changes by immunohistochemistry. An increase in Ktrans and Kep was observed after bevacizumab treatment on days 2 and 4. Similar trends were observed for D^*^ and f on days 2 and 4. However, the parameters of Ktrans, Kep, D^*^ and f were decreased on days 6 and 8. A significant increase of the vessel maturity index between the treatment and control groups was measured on days 2 and 4, but this increase abated by days 6 and 8. IVIM and DCE-MRI are helpful when quantifying the tumor perfusion and evaluating the vascular normalization window after anti-vessel therapy. IVIM and DCE-MRI can outline the important period after anti-vessel treatment.

## INTRODUCTION

In normal physiological processes, such as the menstrual cycle and wound healing, angiogenesis is tightly regulated and creates a balance between pro- and anti-angiogenic factors. However, in tumors, especially malignant tumors, the balance is tilted to promote angiogenesis, causing the development of architecturally and functionally abnormal vasculature [1, 2]. Tumor growth relies mainly on angiogenesis, and the newly formed tumor vessels provide oxygen and nutrients for the growth of tumors. The tumor vasculature is structurally irregular, characterized as being more tortuous, fragile, dilated and disorganized [3, 4]. Tumor vessels are hyperpermeable, and therefore, intravascular fluid and plasma proteins extravasate, leading to an increase in interstitial fluid pressure (IFP). The blood supply is heterogeneous; in some areas, the blood supply is high, and in others, it is low [5]. Abnormal blood supply and interstitial hypertension interfere with the delivery of therapeutics to solid tumors. Hypoxia renders tumor cells resistant to both radiation and a variety of cytotoxic chemotherapy drugs [4]. Consequently, drug delivery to tumors is inefficient, reducing chemotherapeutic efficacy.

The aim of anti-angiogenic therapy is to starve tumor cells by reducing tumor blood vessels, but it has not been as successful as expected. Reducing tumor vasculature makes the tumor more hypoxic and therefore resistant to chemotherapy, allows blood vessels to leak, thus making them prone to tumor cell migration, and reduces the delivery of chemotherapeutic agents [6]. An appropriate dose of anti-angiogenic agents would transiently normalize the tumor vasculature, leading to improving blood perfusion and lower IFP, permitting an increase in chemotherapy agent delivery, thus improving the outcome of the therapy [7]. Bevacizumab, an antibody to vascular endothelial growth factor (VEGF), has been investigated as a targeted mechanism to normalize vasculature in both preclinical and some clinical studies [8, 9]. The functional mechanisms of bevacizumab, via VEGF inhibition, include regression of existing microvessels to impede tumor growth, relative normalization of the surviving mature tumor vasculature, and inhibition of new vessel growth [10]. In fact, many preclinical and clinical studies have shown that anti-angiogenic therapy results in vascular normalization. The period when the vessel normalization is present is called the ‘normalization window’. Normalizing tumor vessels is hypothesized to restore perfusion, thus reducing metastasis and enhancing drug delivery [6]. Some studies used anti-angiogenic drugs in combination with chemotherapy to enhance chemotherapeutic efficacy in multiple cancer types, such as glioblastoma and metastatic colorectal cancer [5, 11]. The efficacy of combined anti-angiogenic and chemotherapy treatment is schedule-dependent. If this normalization window were identified for individual patients, radiation or chemotherapy in this golden period window would maximally improve the effectiveness of the treatment.

Multiparametric magnetic resonance (MR) imaging, which can reflect both the morphologic and the functional changes of the tumors, may be a promising tool to monitor the vascular normalization window. Dynamic contrast-enhanced (DCE) MRI, which uses contrast agents that penetrate into highly permeable tumor vasculature, is now routinely used in both clinical and experimental pharmacological studies and is particularly suitable for the evaluation of agents targeted to tumor vasculature [12, 13]. Several studies have demonstrated that intravoxel incoherent motion (IVIM) diffusion-weighted (DW) imaging may also be promising in this regard, as it can be used to separately evaluate both the molecular diffusivity and the micro-capillary perfusion of tissue [14, 15]. Development of a noninvasive biomarker to investigate the vascular normalization window *in vivo* would be helpful to provide an optimized treatment for malignant tumors. Ideally, this biomarker would be applicable in clinical practice, providing a quantitative measurement that could be objectively used to characterize and monitor the vascular normalization window. In this study, we investigated DCE and IVIM MR imaging parameters in longitudinal settings for monitoring the glioma vascular normalization window after anti-angiogenic therapy.

## RESULTS

**Figure 1:**
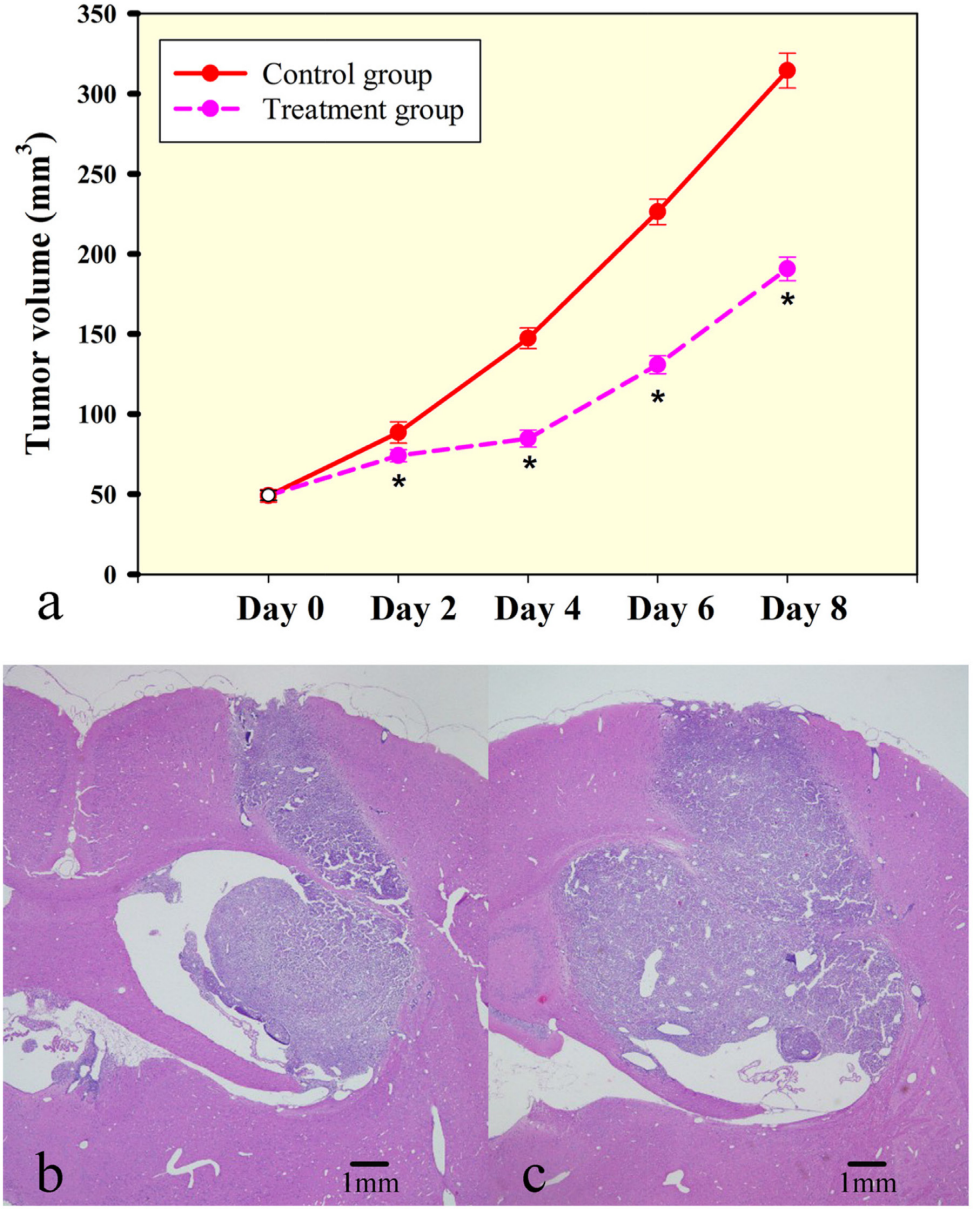
Rat C6 tumor growth is slowed by low dose of bevacizumab treatment **(a)** Bevacizumab treated tumors were significantly smaller compared to control tumors. Tumor volumes were significantly different at four time points. Significance between treatment group and control group was assessed at p<0.05 and indicated by ^*^. (**b** and **c**) Tumor volume increased nearly twice from day 4 to day 8 in treatment group and there was not necrosis in the tumor center. (Hematoxylin and eosin staining, magnification 20X).

Established rat C6 gliomas were divided into a bevacizumab treatment group (n=32) and a control group (n=8) approximately 10 days after tumor cell injection, when tumors were approximately 49.25±1.10 and 49.00±1.38 mm^3^ (P=0.749). Bevacizumab-treated tumors were significantly smaller compared to control tumors on day 2 (74.00±1.34 vs. 88.50±2.34 mm^3^, P<0.001), day 4 (84.63±1.87 vs. 147.25±2.27 mm^3^, P<0.001), day 6 (130.75±1.98 vs. 226.25±2.81 mm^3^, P<0.001) and day 8 (190.63±2.63 vs. 314.38±3.84 mm^3^, P<0.001), demonstrating that a single dose of bevacizumab alone could significantly restrict further growth of established orthotopic C6 glioma xenografts (Figure 1a). There was no necrosis in the tumor center with the tumor growth after bevacizumab treatment (Figure 1b and 1c). For the DCE and IVIM analyses, the reproducibility was tested by comparing a repeated scan. Good measurement repeatability of both IVIM and DCE parameters was determined. IVIM and DCE-MRI parameters in the control group showed good reproducibility of IVIM parameters, with within-subject ICCs of 0.920 for D^*^, 0.831 for f and 0.836 for D and good reproducibility of DCE parameters, with within-subject ICCs of 0.936 for Ktrans, 0.928 for Kep, 0.844 for Ve and 0.837 for Vp.

**Figure 2:**
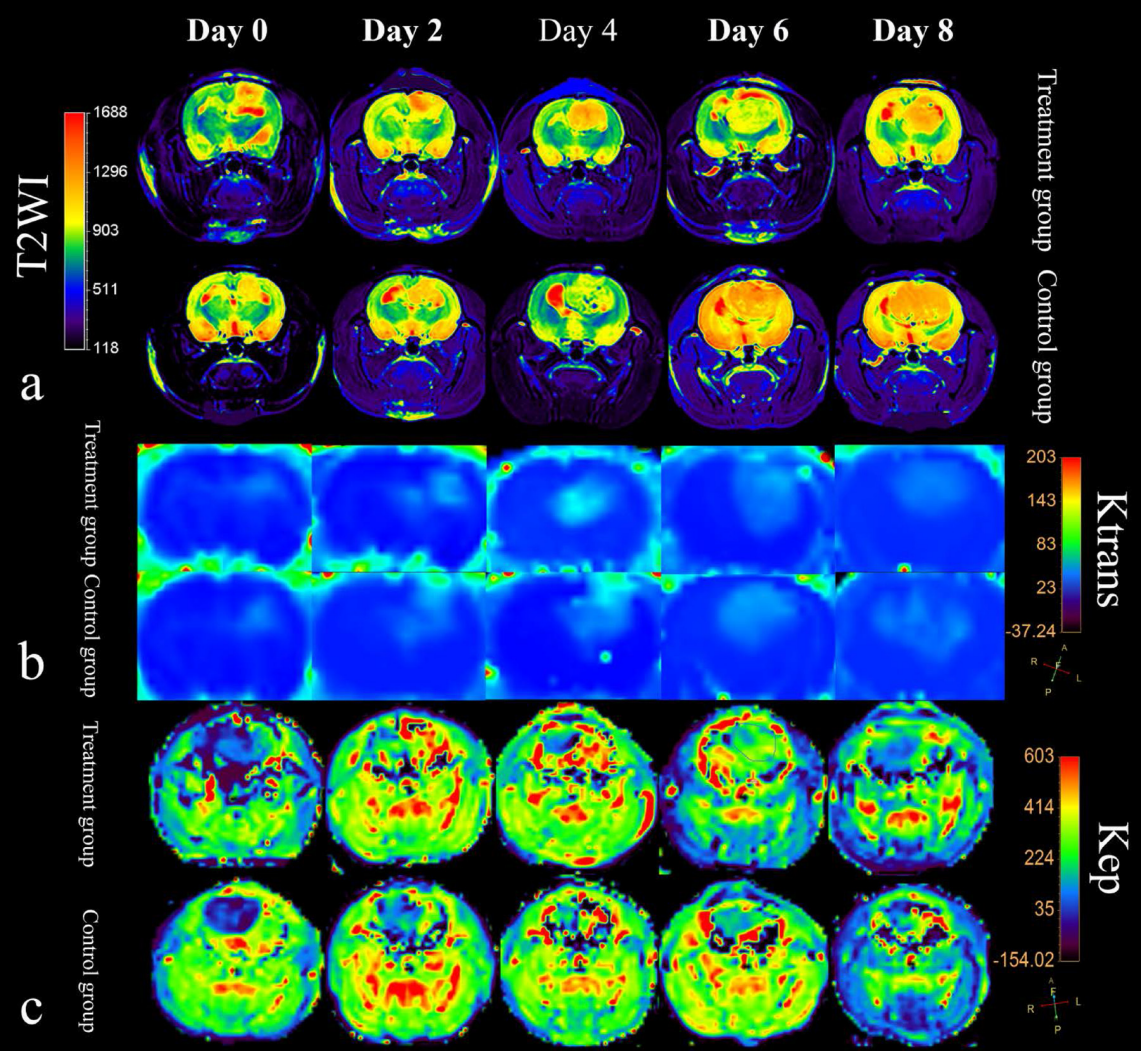
MRI features of rat C6 glioma model in treatment group and control group **(a)** T2-weighted imaging with color overlays. Tumor volume increased as tumor developed both in the treatment group and control group. The extent of peritumoral edema (red signal around tumor) in the treatment group was lower than control group after bevacizumab administration. (**b)** and (**c**) Color maps for DCE-MRI Ktrans and Kep of tumors from day 0 to day 8.

Representative vascular kinetic parameter maps are shown as color overlays in Figure 2. Tumor volume increased as tumors developed both in the treatment group and control group. The extent of peritumoral edema in the treatment group was lower than controls after bevacizumab administration on day 2 and 4, but with the tumor growth, peritumoral edema could be observed again on day 6 and 8 (Figure 2a). Ktrans and Kep in tumor tissues demonstrated with high perfusion in pseudo-color images and increased with tumor growth (Figure 2b and 2c). Ktrans and Kep reached the highest level on day 4 after treatment (56.71±2.11 and 378.94±5.67 min^−1^, respectively).

**Figure 3:**
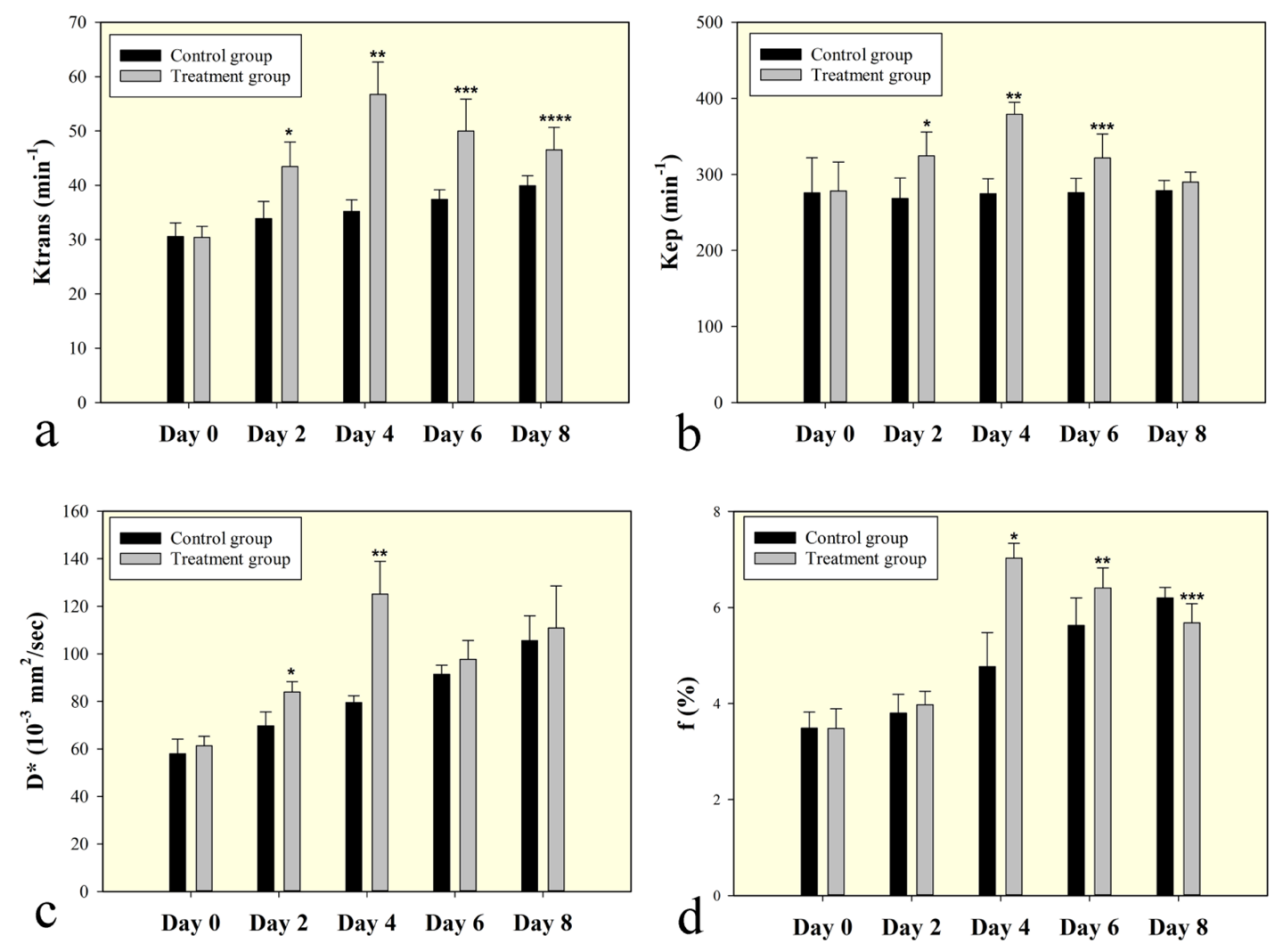
Bar graphs change in perfusion related DCE and IVIM MRI parameters for the treated group shown together with the control Ktrans and Kep steadily increased after bevacizumab treatment on day 2 and day 4. Similar trends were observed for D^*^ and f on day 2 and day 4. Ktrans, Kep, D^*^ and f were decrease when compared to control groups on day 6 and day 8. Symbols above individual bars indicate statistical significance vs the control group (P<0.05).

Serial measurements of DCE-MRI parameters and perfusion-related IVIM-MRI parameters of C6 glioma xenografts in the control and treated group before and after treatment are summarized in Tables 1 and 2. The increase in Ktrans and Kep was observed after bevacizumab treatment on day 2 (P< 0.001 for Ktrans, P< 0.001 for Kep) and day 4 (P< 0.001 for Ktrans, P<0.001 for Kep), with a significant difference when compared to control groups (Figure 3a and 3b). Similar trends were observed for D^*^ and f on day 2 (P=0.002 for D^*^, P=0.075 for f) and day 4 (P<0.001 for D^*^, P<0.001 for f) (Figure 3c and 3d). However, the parameters of Ktrans, Kep, D^*^ and f were decreased when compared to control groups on day 6 (P< 0.001 for Ktrans, P<0.001 for Kep, P=0.107 for D^*^) and day 8 (P< 0.001 for Ktrans, P=0.069 for Kep, P=0.501 for D^*^) (Figure 3). The parameters of Ve, Vp and D did not show significant differences between treatment groups and control groups at all time points. In the control group, Ktrans, f and D^*^ were gradually increased during tumor growth, and the changes were small.

**Table 1:** DCE-MRI parameters of C6 glioma xenografts in the control group and treated group

DCE MRI parameter	Control group	Treated group	T value	P value
**Ktrans(min** ^−1^ **)**				
Baseline	30.53±0.90	30.41±0.71	0.314	0.763
2 days	33.88±1.11^*^	43.43±1.60^*^	−7.123	<0.001
4 days	35.19±0.75^*#^	56.71±2.11	−11.401	<0.001
6 days	37.44±0.61^#^	49.98±2.09^#^	−5.610	<0.001
8 days	39.93±0.65	46.51±1.47^*#^	−4.762	<0.001
Note. Control group, ^*^p=0.269, ^#^p=0.061, treatment group, ^*^p=0.203, ^#^p=0.152, others, p<0.05.				
**Kep(min** ^−1^ **)**				
Baseline	275.70±16.29	278.20±13.47^*^	−3.23	0.756
2 days	268.45±9.50	324.3913±11.09^#^	−5.361	0.001
4 days	274.73±6.96	378.94±5.67	−16.364	<0.001
6 days	275.98±6.62	321.55±11.20^#^	−5.921	0.001
8 days	278.48±4.78	289.87±4.66^*^	−2.144	0.069
p>0.05 ^*^p=0.407, ^#^p=0.839, others, p<0.05.				
**Ve (%)**				
Baseline	128.84±13.40	129.90±14.60	−0.043	0.967
2 days	58.26±8.24	130.22±37.92	−1.69	0.135
4 days	91.00±23.38	116.79±24.68	−1.539	0.168
6 days	100.74±32.67	61.64±9.52	1.304	0.233
8 days	106.68±35.98	131.06±30.84	−0.532	0.611
	p>0.05	p>0.05		
**Vp**				
Baseline	2.78±0.24	3.08±0.38	−0.613	0.559
2 days	2.51±0.11	2.44±0.32	0.197	0.849
4 days	2.30±0.26	2.94±0.41	0.112	0.914
6 days	3.34±0.46	2.47±0.12	1.685	0.136
8 days	2.65±0.30	3.04±0.25	−0.685	0.416
	p>0.05	p>0.05		

**Table 2:** Perfusion-related IVIM MRI parameters of C6 glioma xenografts in the control group and treated group

IVIM MRI parameter	Control group	Treated group	T value	P value
**D (10**^−3^ **mm**^2^**/sec)**				
Baseline	0.78±0.03	0.77±0.03	0.141	0.892
2 days	0.77±0.04	0.77±0.04	−0.037	0.972
4 days	0.71±0.03	0.76±0.03	−1.065	0.322
6 days	0.73±0.03	0.71±0.03	0.838	0.430
8 days	0.76±0.01	0.75±0.04	0.275	0.791
	p>0.05	p>0.05		
D^*^(10^−3^ **mm**^2^**/sec)**				
Baseline	57.96±2.17	61.29±1.40	−1.16	0.283
2 days	69.71±2.08	83.97±1.55	−4.904	0.002
4 days	79.50±1.02	125.09±4.90	−9.396	<0.001
6 days	91.43±1.36	97.75±2.79	−1.849	0.107
8 days	105.57±3.70	110.88±6.26	−0.710	0.501
	P<0.05	P<0.05		
**f (%)**				
Baseline	3.49±0.12^*^	3.48±0.15	0.044	0.966
2 days	3.80±0.14^*^	3.97±0.10	−2.088	0.075
4 days	4.77±0.25	7.03±0.11	−7.846	<0.001
6 days	5.63±0.20	5.41±0.15	−3.100	0.017
8 days	6.20±0.08	5.68±0.14	3.259	0.014
	^*^p>0.05, others, p<0.05	p>0.05		

**Figure 4:**
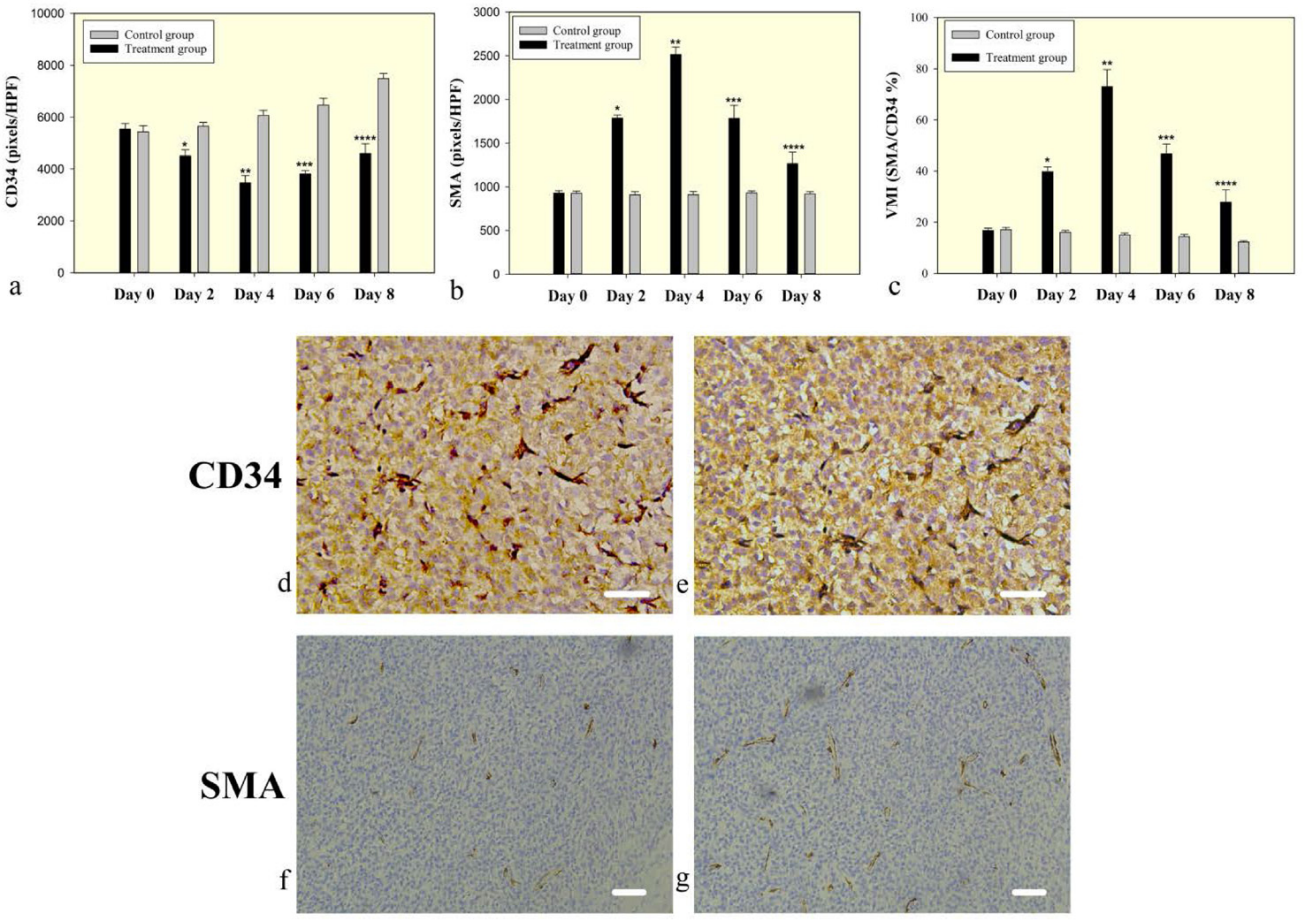
Microvessel density and pericyte coverage in C6 glioma **(a)** CD34 staining for vascular endothelial cells reflects microvessel density. A significantly decreased microvessel density in the bevacizumab treated tumors on days 2, 4, and 6, but slowly increased on day 6 and 8. **(b)** SMA staining for vascular pericyte coverage. **(c)** The vessel maturity index is the ratio of perivascular cells to endothelial cells. A significant increase in VMI in the treated groups was measured on days 2 and 4, but abated by day 6 and 8. Symbols above individual bars indicate statistical significance vs the control group (P<0.05). **(d-g)** Immunohistochemical analysis representative examples of tumors on day 4 after bevacizumab treatment showed that a low density of microvasculature (CD34) and highly covered with perivascular cells (SMA) in the treatment group (e and g) on day 4 compared with control group (d and f). Images were acquired at 200× (d & e) or 100× (f & g) magnification. Scale=50μm.

At the baseline (day 0), there was no significant difference between the bevacizumab-treated and the control tumors in microvessel density (5541.00±76.15 vs. 5438.1250±81.17 pixels/HPF, P=0.118) and in α-SMA staining (928.13±9.12 vs. 925.38±9.01 pixels/HPF, P=0.762). A subsequent significant decrease in microvessel density was observed in the bevacizumab-treated tumors compared to controls on day 2 (4505.00±83.87 vs. 5650.75±52.06 pixels/HPF, P<0.001) that persisted on day 4 (3465.13±101.61 vs. 6064.25±68.83 pixels/HPF, P<0.001). However, the microvessel density was increased on day 6 (3811.38±43.78 vs. 6462.88±93.72 pixels/HPF, P<0.001) and day 8 (4601.63±130.62 vs. 7485.50±70.84 pixels/HPF, P<0.001) (Figure 4a). The α-SMA staining was observed to increase in the treatment groups compared to controls on day 2 (1785.50±12.74 vs. 907.63 ±12.84 pixels/HPF, P<0.001) and day 4 (2514.00±30.43 vs. 909.50±12.81 pixels/HPF, P<0.001), but decrease on day 6 (1782.88±52.85 vs. 930.25±7.34 pixels/HPF, P<0.001) and day 8 (1264.88±46.87 vs. 917.25±9.30 pixels/HPF, P<0.001) (Figure 4b). A significant increase in the vessel maturity index (VMI) was seen in the bevacizumab-treated group, as measured on days 2 (39.71±0.67 vs. 16.07±0.26, P<0.001) and 4 (73.00±2.37 vs. 15.01±0.28, P<0.001). However, this effect had abated by day 6 (46.79±1.34 vs. 14.42±0.28, P<0.001) and day 8 (27.81±1.72 vs. 12.26±0.17, P<0.001), demonstrating that by day 6, some of the relatively mature vessels were undergoing spontaneous regression (Figure 4c). The increase of VMI was due to the increase of pericytes and the decrease of endothelial cells (Figure 4d–4g).

**Figure 5:**
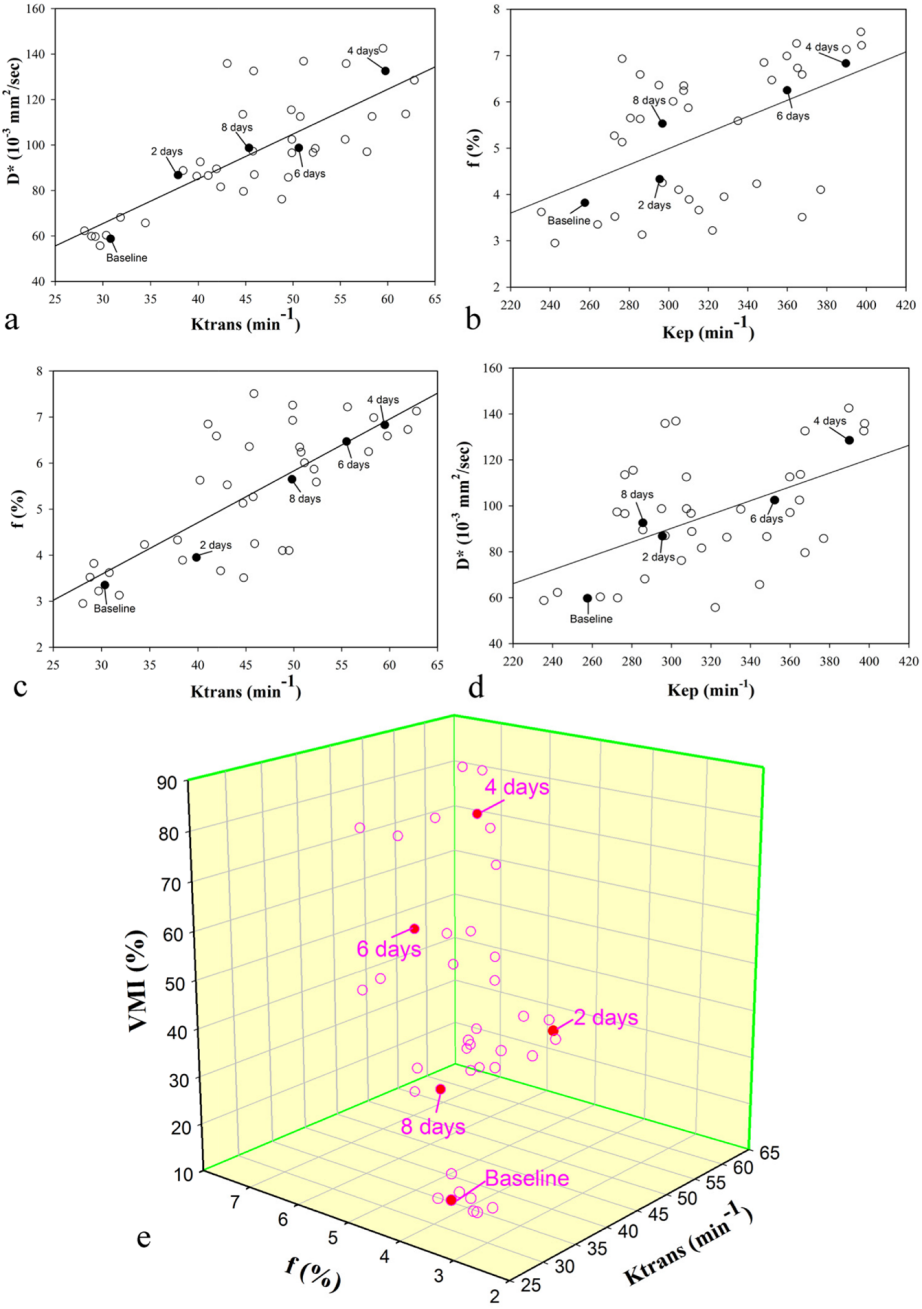
Correlation between DCE and IVIM MRI perfusion parameters and vessel maturity index (VMI) in the treatment group after bevacizumab administration A single representative subject is marked with filled circle on each scatterplot at every time point (day 0, 2, 4, 6 and 8 after treatment). Significant positive correlations (P< 0.05) are observed between **(a)** Ktrans and D^*^, **(b)** Kep and f, **(c)** Ktrans and f, and **(d)** Kep and D^*^. The thick full lines are the linear regression fits to the data. **(e)** 3D scatterplot shows Ktrans and f are positively related with VMI (P<0.05).

Correlations between parameters derived from DCE-MRI (Ktrans and Kep) with parameters derived from IVIM-MRI (D^*^ and f) and the vessel maturity index (VMI) are listed in Table 3. In the bevacizumab treatment group, both Ktrans and Kep showed a significant positive correlation with D^*^ and f, respectively (D^*^: r=0.792, P<0.001 and r=0.541, P<0.001; f: r=0.775, P<0.001 and r=0.537, P<0.001) (Figure 5a–5d). In addition, perfusion-related IVIM parameters (D^*^ and f) and DCE parameters (Ktrans and Kep), showed a significant positive correlation with VMI (P<0.001) (Figure 5e).

**Table 3:** Correlation of perfusion-related IVIM-MRI parameters with DCE-MR parameters and vessel maturity index (VMI) in the bevacizumab treatment group

MRI parameters	Ktrans (min^−1^)		Kep (min^−1^)		VMI (%)	
	Coefficient (r)	P value	Coefficient (r)	P value	Coefficient (r)	P value
D^*^(10^−3^ mm^2^/sec)	0.792	<0.001	0.541	<0.001	0.681	<0.001
f (%)	0.775	<0.001	0.537	<0.001	0.746	<0.001
VMI (%)	0.779	<0.001	0.749	<0.001	-	-

Note. Significant (p < 0.05) correlation.

## DISCUSSION

In this study, we have shown that functional MR imaging parameters can predict the vascular normalization window of an orthotopic glioma model in rats after anti-angiogenic therapy. Although the importance of normalization of the intratumoral vasculature is becoming increasingly appreciated [6, 16, 17], this is the first study to show this normalization window’s beginning and end time points using noninvasive MR imaging biomarkers.

Biopsy is the standard to evaluate tumor angiogenesis, but the localization of the puncture site and the invasiveness of the puncture operation itself demonstrate that this method cannot dynamically evaluate the panorama of tumors [18]. Thus, a noninvasive imaging means must be developed. DCE-MRI perfusion imaging has been widely used in diagnosis and therapeutic effectiveness evaluation. Quantitative parameters can be calculated with DCE-MRI on different pharmacokinetic models (Ktrans, which reflects vessel permeability, Kep, regurgitation of contrast medium, and Vp, plasma volume) [19, 20]. These perfusion parameters have been used as indicators for the clinical evaluation of anti-tumor drugs. Previous studies have demonstrated that these parameters are well correlated with microvessel density and can be used to distinguish the differences in glioma grading and evaluate neovascularization patterns [21, 22]. The IVIM applies bi-exponential fitting to signal decay obtained with multiple b-values and then separates molecular diffusion and micro-capillary perfusion [23]. The IVIM can simultaneously acquire diffusion and perfusion parameters, which reflect tumor cellularity and vascularity, respectively [24]. Moreover, IVIM does not require intravenous contrast agent injection for the data acquisition. Our results demonstrated that perfusion parameters from both IVIM and DCE increased significantly on 2- and 4-day follow-up in the bevacizumab-treated group, peaked on day 4 and partially decreased on 6- and 8-day follow-up.

Anti-angiogenic therapies change the density and architecture of the tumor vasculature network and create a transient window of vascular normalization shortly after anti-angiogenic therapy begins [25]. Vascular normalization repairs the morphological structure and function of the tumor vessel wall and tumor microvessel network and can improve tumor perfusion and oxygenation [4, 26]. The efficacy of chemotherapy and radiotherapy may be enhanced during the transient window due to increased perfusion and decreased vascular permeability [25]. We have shown that abated tumor microvessel density via bevacizumab produce a transient remodeling of the intratumoral vasculature, which creates a period of time during which there is improved intratumoral perfusion in rat glioma xenografts. This remodeling of the vasculature to a more mature, functional phenotype seems to have been achieved by destroying the immature vessels in which the endothelial cells lack the support of adjacent pericytes [17]. In our study, the tumor vessel maturity index (VMI) increased after bevacizumab, reached the peak on day 4, and then decreased gradually. Moreover, those perfusion-related MRI parameters had a positive correlation with VMI after bevacizumab treatment. Sorensenet al.[27] found that the vascular normalization index (VNI), derived from combing with MRI biomarkers (Ktrans and cerebral blood volume) and circulating collagen IV, is associated with disease progression and overall survival in recurrent glioblastoma patients.

Previous experiments have shown that aberrant tumor vessels are reversed as early as 24-72 h after the onset of anti-angiogenic therapy and are sustained for different periods, from a few to a dozen days or sometimes longer [5, 28]. Other studies also showed that the appearance of a vascular normalization display usually lasts for only 1-2 days after the start of treatment, providing a narrow window for enhanced drug delivery [29, 30]. The window of increased perfusion from vascular normalization depends on the dose and potency of anti-angiogenic therapy [31]. In our study, we used a single low dose of bevacizumab. The tumor vascular normalization appeared on day 2 and gradually faded away on day 6 after anti-angiogenic treatment. The normalization window time lasted approximately 4 days. Some studies also reported that lower doses might improve perfusion and outcome [32, 33]. Huang et al. [33] found that low doses of an anti-VEGFR2 antibody (10 or 20 mg/kg) increased perfusion compared with a high dose (40 mg/kg) or control immunoglobulin G (IgG) in a breast cancer model.

The DCE and IVIM MR parameters may help monitor the effectiveness of anti-angiogenic therapy because the normalized vessels are characterized by VMI, which is one of the pathologic hallmarks in the process of anti-angiogenic therapy. A previous study demonstrated that D^*^ and f showed significant correlation with microvessel density, but D did not [34]. Our study results showed that perfusion-related IVIM parameters (D^*^ and f) had a significant correlation with DCE parameters (Ktrans and Kep) (P<0.05). Joo et al. [14] revealed a good correlation between D^*^ and f with Ktrans and iAUC (initial area under the gadolinium concentration-time curve until 60 seconds) in a rabbit liver tumor model for longitudinal monitoring of vascular disrupting therapy. Since iAUC is a semiquantitative DCE-MRI parameter, we did not conduct it in this study.

There are some limitations in our study. First, we used bevacizumab in our experiment, which is a recombinant humanized monoclonal antibody. This humanized bevacizumab may be less effective in rats because of its lack of specificity. The rat antibody analog may be a better option. This deficiency will be improved by using rat-derived VEGF antibody in future research. Second, the present study is limited to a single tumor type, and the analysis did not take into consideration tumor heterogeneity that may potentially skew the results. Third, we used only one kind of anti-angiogenic drug and a single dose to study. In the next study, different types and doses of anti-angiogenic drugs may be used to make a comparative study.

In conclusion, IVIM and DCE MRI show great potential in the noninvasive assessment of the normalization window induced by bevacizumab. The noninvasive MRI biomarkers can be useful tools to monitor the temporal vascular changes in tumors after anti-angiogenic therapy and can outline the important period of vascular normalization window.

## MATERIALS AND METHODS

### Cells

The C6 rat glioma cells were cultured in a CO_2_ incubator in an atmosphere with 6% CO_2_ and 90% humidity for 3 days in DMEM (Gibco, Carlsbad, CA, USA) containing glucose supplemented with 10% bovine fetal serum, 2 mM glutamine and 1% penicillin-streptomycin. Before experiments, cells were harvested in the log phase of growth; their viability was determined by 0.4% trypan blue exclusion and then trypsinized to obtain the cell suspension for implantation.

### Model of intracranial C6 glioma in rats

Animals were treated in accordance with the guidelines approved by the Animal Ethical Committee. Adult Sprague Dawley rats (180-220 g) were obtained from the Animal Experiment Center of our university; they were housed in a standard animal facility under 12-hour light and 12-hour dark conditions and allowed free access to standard food and water. All experimental rats were anesthetized by an intraperitoneal injection of 10% chloral hydrate (0.3 ml/100 g). The body temperature was maintained approximately 37.5°C with a feedback-controlled heating pad connected to a rectal probe. Rats were placed in a stereotaxic head holder (RWD life science, Shenzen, China), and then a midline scalp incision was performed and the calvarium was exposed. The skull was drilled with a micro cranial drill to produce a 1 mm hole allowing the insertion of a microsyringe. The C6 glioma cell suspension (1×10^6^ cells in 10 μl PBS) was slowly injected into the right brain parenchyma (coordinates relative to bregma: 3.0 mm lateral and 6.0 mm deep) according to the rat stereotaxic atlas (Paxinos and Watson, 2006). The tumor cells were injected constantly at a rate of 0.5 μL/min until the entire volume was injected. The needle was left in place 5 min after the end of the injection and then slowly removed. The scalp was sutured, and the rats were allowed to recover from anesthesia.

### MRI scan

**Figure 6:**
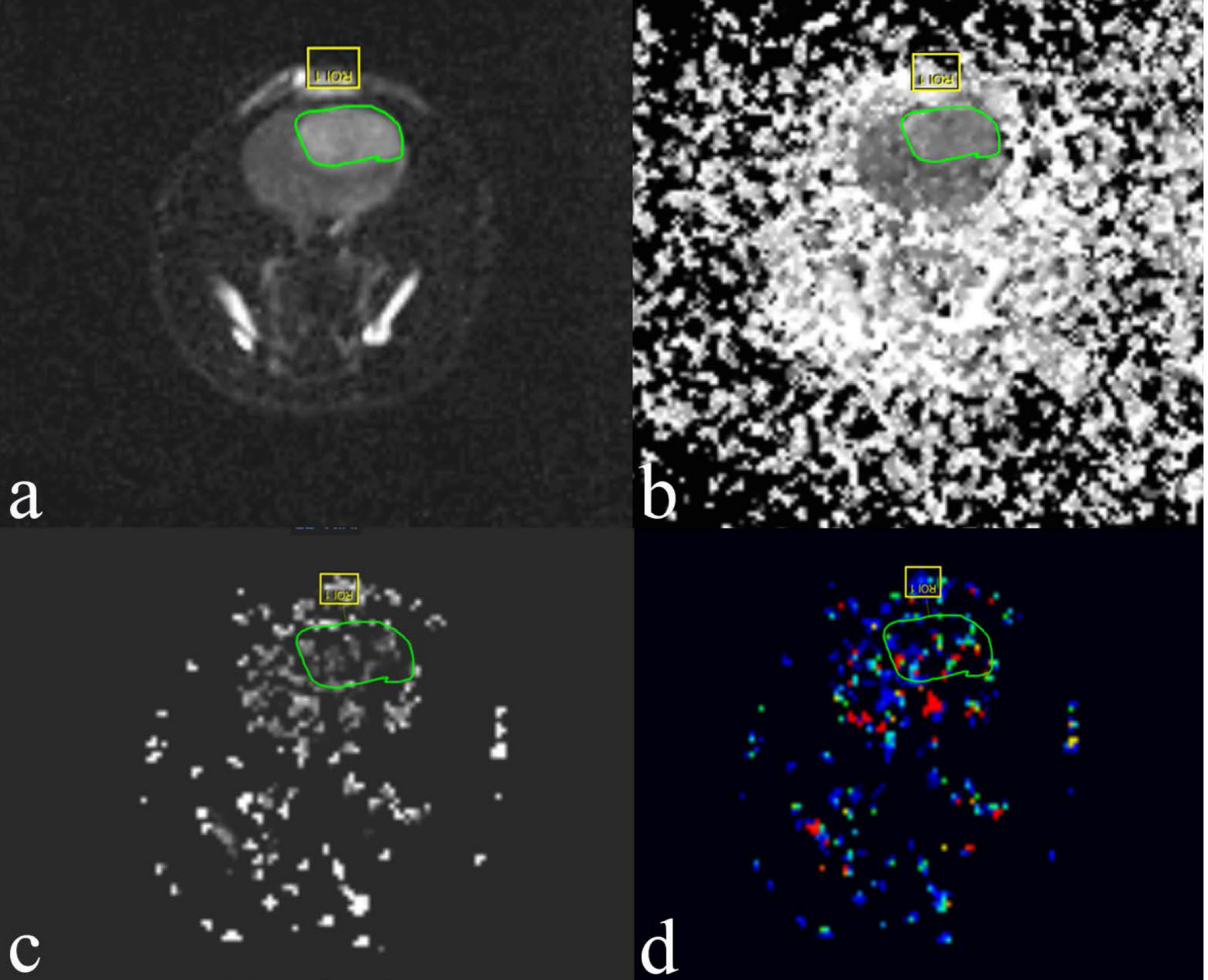
Examples of the ROIs (region of interest, green circle) selected for a single slice through the tumor grown in rat C6 glioma 4 days posttreatment with bevacizumab The images are of IVIM. **(a)** Diffusion-weighted image with b = 0 s/mm^2^. **(b)** Pure molecular diffusion (D). **(c)** Perfusion-related diffusion (D^*^). **(d)** Perfusion fraction (f).

The MR examination was done using a 3.0-T clinical MRI system (Philips Ingenia, Netherlands) with a two-channel phased-array coil (50 mm in diameter; Philips) to obtain all MR images. The rats were anesthetized and kept warm on a heating pad circulating with water at 37.5°C. The rat’s head was placed inside the coil in the prone position, and the head was fixed to prevent movement. IVIM-MRI was performed before injection of contrast agent. IVIM-MRI was acquired in the coronal plane using a 2D EPI sequence with spectral attenuated inversion recovery (SPAIR) fat suppression, a 120x120 matrix, TR/TE (repetition time and echo time) = 788/94 ms, b-values = 0, 20, 50, 70, 90, 110, 130, 150, 200 and 500 s/mm^2^ and 3 directions. The acquisition time for each DWI was 8 min 25 sec. The DCE-MR protocols were the following: two-dimensional T1WI single-shot Turbo Field Echo sequence (TR = 4.8 ms; TE = 2.4 ms; NSA= 4; field of view, 40 mm × 40 mm; matrix size, 320 × 320; slice thickness, 2 mm; flip angle = 8°; and slice number = 5). The acquisition time for each T1-weighted image set was 6 min 20 sec. Following the first 5 images scanning, a bolus dose of gadolinium-DTPA-BMA (GADODIAMIDE, GE Healthcare Pharmaceuticals, 0.2 mmol/kg, diluted 1:10 from 0.5 mmol/mL in 0.9% saline solution) was administered via tail-vein catheter at a rate of 3 ml/min. The IVIM behavior of the diffusion-weighted signal SI was described by a biexponential model according to Le Bihan et al [35]. SI=SI_0_[(1 − f) ^*^exp (−b^*^D) + f ^*^exp (−b^*^D^*^)] where D is the diffusion coefficient, D^*^ is the pseudodiffusion coefficient, and f is the perfusion fraction. SI and SI_0_ are the signal intensity at a given b-value and at b= 0 s/mm^2^, respectively. The DW imaging data sets acquired with multiple b values were post processed by using an open source software program to extract IVIM parameters, including f (the perfusion fraction), D (the true diffusion coefficient) and D^*^ (the pseudodiffusion co-efficient) (Figure 6). Mean values of those parameters were measured by drawing a region of interest (ROI) outlining the tumor border on the largest cross section of the tumor.

### Bevacizumab treatment

Rats were given one vein injection dose of 10 mg/kg bevacizumab (Avastin, F. Hoffman-La Roche, Switzerland) when the tumors reached approximately 3∼5mm in diameter. Tumor volume was calculated as: V = (π/6)×a×b×c (mm^3^), where a, b and c are the three orthogonal diameters measured in MRI. The control group received saline only in the same dose. Control and treatment groups were evaluated on days 0, 2, 4, 6 and 8.

Histology rats were euthanatized at each of the time points on days 2, 4, 6, and 8 after bevacizumab administration. Each brain was fixed in 4% neutral formalin and cut into 4-μm-thick coronal slices after dehydration and paraffin embedding. Hematoxylin-eosin (H&E) staining, CD34 and α-smooth muscle actin (α-SMA) immunohistochemical staining were then performed on these slices. Microvessel density (MVD) was determined using CD34 staining. Three fields per tumor sample were digitally photographed at 200X, focusing on endothelial cell “hotspots” without tumor necrosis. Positive staining was then quantified employing Image-Pro Plus 6.0 software (Media Cybernetics, Rockville, USA) and reported as the number of pixels per high power field (HPF). The vessel maturity index (VMI) was calculated as the ratio of α-SMA to CD34 staining.

### Statistical analysis

The data were presented as the mean± standard error of the mean (SEM) unless otherwise stated. Statistical comparisons were made using Student’s t-test and one-way analysis of variance. Differences between groups were evaluated by the LSD test and Student-Newman-Keuls test. Correlations between IVIM and DCE MRI parameters were also investigated by calculating Pearson correlation coefficients at baseline and day 2, day 4, day 6 and day 8. Differences were considered statistically significant at p values <0.05. The statistical analysis was performed using SPSS 23.0 software (IBM Corporation, New York, USA). MRI scanning was performed at baseline and at 8-hour follow-up in the same session to assess the reproducibility of IVIM and DCE MR parameters. Reproducibility of all parameters was then evaluated using intraclass correlation coefficients (ICC). Within the 95% confidence interval (CI), ICC ≥0.75 was considered to represent good agreement [36, 37].
