# Correction: MR imaging biomarkers evaluating vascular normalization window after anti-vessel treatment

**DOI:** 10.18632/oncotarget.28217

**Published:** 2022-05-05

**Authors:** Jun Yang, Chengde Liao, Yifan Liu, Guangjun Yang, Tengfei Ke, Yingying Ding, Qinqing Li

**Affiliations:** ^1^Department of Radiology, The Third Affiliated Hospital of Kunming Medical University, Yunnan Cancer Hospital, Kunming 650118, Yunnan, P.R. China

**This article has been corrected:** In Figure 4, panel ‘E’ contains an accidental duplication of panel ‘D’. The corrected Figure 4 (panels ‘D’ through ‘G’), is shown below. The authors declare that these corrections do not change the results or conclusions of this paper.

Original article: Oncotarget. 2018; 9:11964–11976. 11964-11976. https://doi.org/10.18632/oncotarget.22600

**Figure 4:**
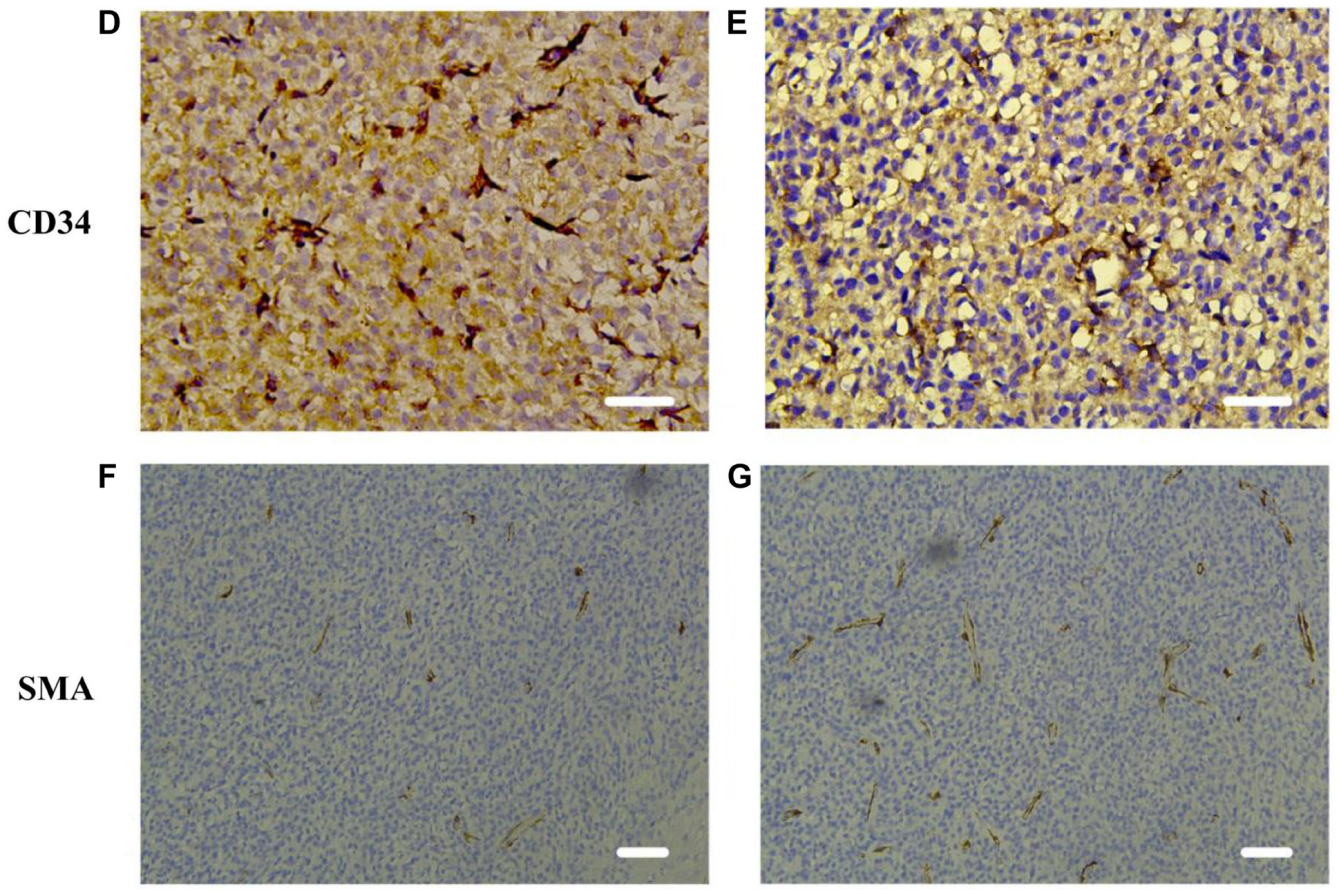
Microvessel density and pericyte coverage in C6 glioma. (**D**–**G**) Immunohistochemical analysis representative examples of tumors on day 4 after bevacizumab treatment showed that a low density of microvasculature (CD34) and highly covered with perivascular cells (SMA) in the treatment group (E and G) on day 4 compared with control group (D and F). Images were acquired at 200× (D, E) or 100× (F, G) magnification. Scale = 50 μm.

